# Influence of observable and unobservable exposure on the patient's risk of acquiring influenza-like illness at hospital

**DOI:** 10.1186/2047-2994-4-S1-O60

**Published:** 2015-06-16

**Authors:** C Payet, N Voirin, P Vanhems, R Ecochard

**Affiliations:** 1 Hospices Civils De Lyon, Universite Lyon, Lyon, France

## Introduction

During outbreaks of hospital-acquired influenza-like illness (HA-ILI) healthcare workers, patients, relatives, and visitors are each source of infection for the others. Quantifying the contribution of various exposures will help improve prevention and control of HA-ILI outbreaks.

## Objectives

The objective was to study the influence of observable exposure to contagious patients and HCWs and of unobservable exposure to other sources on the patient's risk of acquiring influenza-like illness at hospital.

## Methods

On the basis of data from three influenza outbreaks at hospital, we used a statistical model and Bayesian inference to estimate the attributability of HA-ILI to each of: 1) exposure to recorded *vs.* unrecorded sources; 2) exposure to contagious patient *vs.* contagious healthcare workers; 3) exposure during observable *vs.* unobservable contagious period of the recorded sources; and, 4) the moment of exposure.

## Results

Among recorded sources, 59% (95% credible interval: 34-83%) of HA-ILIs of patients were associated with exposure to contagious patients and 41% (17-66%) with exposure to contagious healthcare workers. Exposure during the unobservable contagiousness period of source patients and healthcare workers accounted for 49% (19-75%) and 82% (51-99%) of HA-ILIs, respectively. About 80% of HA-ILIs were associated with exposure one day earlier.

## Conclusion

Secondary cases of HA-ILI might appear as soon as the day after the detection of a primary case highlighting the explosive nature of HA-ILI spread. Unobservable transmission was the main cause of HA-ILI outbreaks suggesting that symptom-based control measures alone might not reduce transmission. The results support vaccination against influenza of patients and healthcare workers and rapid interventions to control transmission.

## Disclosure of interest

None declared.

